# Comment: Isolation and Screening of Bacteria for Their Diazotrophic Potential and Their Influence on Growth Promotion of Maize Seedlings in Greenhouses

**DOI:** 10.3389/fpls.2017.00212

**Published:** 2017-02-28

**Authors:** Jeffrey S. Norman, Jake R. Hare, Maren L. Friesen

**Affiliations:** Plant Biology Department, Michigan State UniversityEast Lansing, MI, USA

**Keywords:** diazotrophs, nitrogen fixation, plant growth promotion, acetylene reduction assay, maize

Recent papers by Kifle and Laing showed that diazotrophs isolated from Maize tissue and Maize-associated soil have the potential to promote growth of this important crop when used to inoculate seeds prior to planting (Kifle and Laing, 2016a,b). The fact that the authors focused their studies on diazotrophs presumes that the biological nitrogen fixation (BNF) is key to the mechanism of plant growth promotion by the microorganisms they tested. However, numerous other studies (e.g., Khalid et al., 2004) have found plant growth promotion by organisms isolated from the rhizosphere that were not screened for BNF, implying that this trait is not necessarily the key mechanism of plant growth promotion. By conducting additional analysis of their data, we find the striking result that rates of acetylene reduction in pure culture presented by Kifle and Laing (2016a) show a strong positive relationship with two metrics of plant health measured in their greenhouse experiment: chlorophyll content (Figure 1A) and stomatal conductance (Figure 1B). The acetylene reduction assay as conducted by Kifle and Laing (2016a) should serve as a proxy for maximum potential rates of BNF achievable by the diazotrophs they investigated. The relationships shown in Figures 1A,B therefore lend credence to the argument that BNF plays an important role in plant growth promotion by the microbes that Kifle and Laing (2016a) tested.

**Figure 1:**
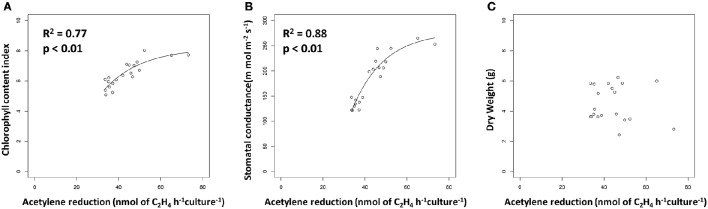
**Metrics of plant growth vs. potential nitrogen fixation, as measured by acetylene reduction, in Maize plants inoculated with diazotrophs**. Data are from Kifle and Laing (2016a). **(A)** Chlorophyll content vs. potential nitrogen fixation. Line represents a significant exponential non-linear regression (y = −1181 + 1458e^−0.6627x^). **(B)** Stomatal conductance vs. potential nitrogen fixation. Line represents a significant exponential non-linear regression (y = −9.516 + 17.71e^−0.0558x^). **(C)** Dry weight vs. potential nitrogen fixation.

By omitting fixed nitrogen sources from their fertilization regime, Kifle and Laing (2016a) designed their diazotroph inoculation experiment to investigate the direct role of BNF in plant growth promotion. Indeed, studies using stable isotope tracers have shown that nitrogen fixed by diazotrophic inoculants was incorporated into plant biomass. For example, Zakry et al. (2012) used the ^15^N pool dilution technique to show that Oil Palm plants inoculated with the endophyte *Bacillus sphaericus UPMB-10* obtained the majority of their nitrogen from fixation after 8 months of growth, while Knoth et al. (2014) found similar results for Poplar plants inoculated with multi-strain diazotrophic consortia. However, bacteria are known to promote plant growth through multiple other mechanisms besides diazotrophy (Friesen et al., 2011) and these mechanisms, which we outline below, may be at least partially responsible for the relationships presented in Figure 1 as well.

Bacteria may increase nitrogen concentrations without diazotrophy through exoezyme release (e.g., Wallenstein and Weintraub, 2008). Rhizosphere bacteria may also enhance plant growth by altering the availability of nutrients other than nitrogen, such as through the release of acids that solublize phosphate (Richardson et al., 2009) or siderophores that bind iron (Alexander and Zuberer, 1991). Many root-associated bacteria can directly alter phytohormones, with wide-ranging effects on plant form and function (Friesen et al., 2011). For example, many bacteria produce auxin or gibberellins that increase root growth (Sirrenberg et al., 2007) and it is common for them to express ACC deaminase that alters plant ethylene levels (Glick et al., 2007). Finally, rhizosphere bacteria can mediate other interactions, these include reductions in the impact of pathogens (Compant et al., 2005) and promotion of colonization by mutualists (Srinivasan et al., 1996). While diazotrophy is not required for many mechanisms of plant growth promotion, BNF could benefit microbes that promote plant growth by ensuring their persistence in the carbon-rich rhizosphere; it is therefore likely that many strains of plant growth promoting bacteria are diazotrophic regardless of their mechanism of plant growth promotion. The authors should consider testing their strain collection for plant growth promotion by non-diazotrophic means to further investigate the mechanisms behind plant growth promotion by diazotrophs in Maize.

Regardless of the mechanism, or combination of mechanisms, by which diazotrophs promote Maize growth through BNF, the increases in chlorophyll content and stomatal conductance shown in Figures 1A,B suggest that these plants respectively increased and CO_2_ uptake and fixation and in response to diazotroph inoculation. Strangely, we found no relationship between dry weight, seemingly the most straightforward plant growth metric measured by Kifle and Laing (2016a), and potential BNF by the diazotrophs used for seed inoculation (Figure 1C). It seems that the increased carbon fixation triggered by diazotroph inoculation had not yet translated to increased Maize biomass in this study. It should be noted that the same authors used a subset of the diazotrophic isolates from this study for field inoculation trials of Maize plants (Kifle and Laing, 2016b); neither dry weight nor cob production, the variable of most agricultural interest, were related to the lab-measured potential diazotrophy of the strains used in that study (data not shown).

While Kifle and Laing (2016a,b) analyzed their data with an eye toward increased crop production using single strain inocula, our regression-based analysis provides insight into the potential role of BNF in plant growth promotion. Interestingly, the positive relationships between plant growth metrics and potential BNF by diazotrophic inoculants shown in Figures 1A,B saturate as potential BNF rates increase, indicating that there is little difference between plant growth promotion potential amongst the highest performing diazotrophs. In fact, the authors found no statistically-significant differences in chlorophyll content in plants inoculated with the strains with the 11 highest rates of potential BNF pure culture, despite the fact that measured rates of acetylene reduction varied nearly two-fold (from 42.0 n mol of C_2_H_4_ h^−1^ culture ^−1^ in strain A2 to 73.2 n mol of C_2_H_4_ h^−1^ culture ^−1^ in strain V9). This trend extended to a 12th strain for stomatal conductance, where there was no significant difference between plants inoculated with the top performer (strain V9) and strain V10, which only showed acetylene reduction rates of 35.3 n mol of C_2_H_4_ h^−1^ culture ^−1^. Future work in this area should focus on increasing the range of potential BNF of diazotrophic strains tested to fully investigate the relationship between plant growth promotion and potential BNF by diazotrophic inoculants.

## Author contributions

JN conceived of the idea for this manuscript, analyzed data, created figures, and contributed text to the final version. JH and MF contributed to the development of the idea for this manuscript, contributed text to the final version, and edited the manuscript.

### Conflict of interest statement

The authors declare that the research was conducted in the absence of any commercial or financial relationships that could be construed as a potential conflict of interest.
